# Methodological pitfalls in indirect treatment comparisons: insights from a recent systematic review and analysis for C3 glomerulopathy

**DOI:** 10.57264/cer-2026-0054

**Published:** 2026-08-09

**Authors:** Brian Hutton, James Fotheringham, Andrew Ervin, Raisa Sidhu, Corey Lourenco, Imtiaz A Samjoo, Chris Cameron, Bridget Dunne

**Affiliations:** 1 Ottawa Hospital Research Institute, Methods & Implementation Research Program, Ottawa, Ontario K1H 8L6, Canada; 2 Faculty of Population Health, School of Medicine & Population Health, University of Sheffield, Sheffield, UK; 3 Novartis Pharmaceuticals UK Limited, London, UK; 4 Novartis Pharma AG, Basel, Switzerland; 5 CRG-EVERSANA Canada Inc., Burlington, Ontario L7N 3H8, Canada; 6 Novartis Ireland Ltd., Ireland

**Keywords:** APPEAR-C3G, C3 glomerulopathy, feasibility assessment, indirect treatment comparison, iptacopan, matching-adjusted indirect comparison, pegcetacoplan, VALIANT

## Abstract

**Aim:**

Indirect treatment comparisons (ITCs), as outlined in NICE and ISPOR guidance, require careful evaluation of cross-trial heterogeneity to ensure valid comparisons, particularly in rare diseases with limited evidence. C3 glomerulopathy (C3G) is an ultra-rare, complement-mediated kidney disease with high unmet need, making appropriate application of ITC frameworks especially critical. This appraisal evaluates the feasibility of applying ITC principles to compare Phase III trials of iptacopan (APPEAR-C3G) and pegcetacoplan (VALIANT) in the absence of head-to-head evidence.

**Materials & methods:**

Feasibility of an ITC in C3G was assessed through critical appraisal of APPEAR-C3G and VALIANT randomized controlled trials, focusing on alignment of eligibility criteria, baseline characteristics and outcome definitions, in line with NICE DSU TSD-18, CHTE2020 and ISPOR guidance. A systematic literature review (SLR) was then conducted to identify published ITCs comparing iptacopan and pegcetacoplan in C3G, which were evaluated for methodological rigor, transparency and credibility according to NICE and ISPOR recommendations.

**Results:**

Substantial heterogeneity was observed between APPEAR-C3G and VALIANT. Overlap was limited to small subpopulations, with imbalances in baseline characteristics, differences in end point reporting, and noncomparable placebo responses. These issues indicate that anchored ITCs are not feasible using currently available data without extensive adjustments that conflict with NICE and ISPOR guidance. The SLR identified one ITC poster with limited methodology comparing these trials. However, when the results were subsequently published in a manuscript, crucial methodological details including justification of effect modifiers, modeling diagnostics, analytic procedures, were still unavailable. Other concerns, such as using standard matching-adjusted indirect comparison methodology in the presence of substantial cross-trial heterogeneity, and the resulting limited ESS observed frequently in rare diseases, were confirmed, thus undermining credibility of conclusions.

**Conclusion:**

ITCs in C3G face significant methodological challenges due to pronounced trial heterogeneity and small sample sizes inherent to this ultra-rare disease. These limitations complicate the conduct and interpretation of arising ITCs, highlighting the need for transparent and methodologically robust approaches. Consequently, payers, decision makers, and HTA bodies should interpret existing C3G ITCs with caution. These findings inform broader application of ITC methods in rare diseases, identifying areas for future evidence generation and analytical innovation.

Indirect treatment comparisons (ITCs) are commonly used when head-to-head randomized trials are unavailable or do not address all relevant comparators. However, the validity of an ITC depends on strong assumptions of similarity between patient populations and study characteristics (e.g., inclusion criteria, outcome definitions, outcome measurement, treatment administration, background therapies, population characteristics and study design) [1]. Even when head-to-head evidence exists, ITCs can play an important complementary role by integrating broader evidence to strengthen comparative assessments. Guidance from the National Institute for Health and Care Excellence (NICE) Decision Support Unit (DSU) [1,2] and the International Society for Pharmacoeconomics and Outcomes Research (ISPOR) Task Force [3,4] stresses the importance of rigorous assessment of cross-trial heterogeneity, transparent reporting of adjustment diagnostics (including effective sample size [ESS] in population-adjusted methods), and careful interpretation of ITCs to inform health technology assessment (HTA) and payer decision-making. Thus, to meet the recommended rigor of these ITC guidance documents, sufficient details regarding methods and findings corresponding to appraisals of cross-trial population similarity and adequacy of model fit, are important considerations for readers who wish to assess the validity of a reported ITC.

C3 glomerulopathy (C3G) is an ultra-rare, complement-mediated kidney disease caused by overactivation of the alternative pathway of the complement system, leading to injury in the glomeruli [5,6]. Globally, there are an estimated 1–2 new cases of C3G per million individuals per year, with a median age of 23 years at diagnosis [7–9]. Despite a similar clinical manifestation to C3G, immune-complex membranoproliferative glomerulonephritis (IC-MPGN) is a disease in which immune complexes deposit in the kidneys and trigger secondary complement activation [10,11]. The incidence of IC-MPGN is unknown and the median age at diagnosis is estimated to be 39 years [12,13]. While different underlying mechanisms underpin C3G and IC-MPGN, similarities in their clinical presentation and rarity have resulted in the two diseases often being considered together in research and in clinical practice. Proteinuria is a central feature of C3G and IC-MPGN and reflects ongoing glomerular injury; increased proteinuria is indicative of disease worsening [10]. A reduction in proteinuria (often measured by UPCR level) indicates treatment response, and has been associated with reduced kidney failure risk [14]. Without effective treatment, both C3G and IC-MPGN often progress to chronic kidney disease and ultimately kidney failure, with approximately 50% of C3G patients progressing to kidney failure within 10 years of diagnosis [5], thus making early changes in proteinuria an important marker of long-term outcomes [14].

Two recent Phase III clinical trials investigated advanced therapeutic options for C3G: iptacopan (a Factor B inhibitor) in APPEAR-C3G (NCT04817618) [15–17], and pegcetacoplan (a C3/C3b inhibitor) in VALIANT (NCT05067127) [18–20]. The APPEAR-C3G trial [15–17] was a multicenter, randomized, double-blind, placebo-controlled, Phase III trial that enrolled adult native C3G patients only, with a 26-week double-blind period followed by a 26-week open-label extension. The VALIANT trial [18–20] was a multicenter, randomized, double-blind, placebo-controlled Phase III study in adolescents and adults with C3G or primary IC-MPGN, including native-kidney and post-kidney-transplant disease. VALIANT had a 26-week double-blind period, followed by a 26-week open label extension.

Given the ultra-rare nature of C3G, direct comparison by head-to-head trials is not feasible, emphasizing the importance of prudent feasibility assessment of indirect comparison. The feasibility and quality of indirect comparison between iptacopan and pegcetacoplan based upon APPEAR-C3G [15–17] and VALIANT [18–20], respectively, depends upon the degree to which the study designs, populations, endpoints and measurement methods of these trials can be aligned, and whether appropriate diagnostics are transparently reported, as outlined by generic NICE and ISPOR ITC guidelines [1–4].

Importantly, no rare disease-specific ITC methodological guidance has been issued by NICE or ISPOR to date. The NICE DSU Technical Support Documents and ISPOR Task Force reports cited throughout this manuscript provide generic methodological frameworks, rather than standards tailored to ultra-rare conditions such as C3G. In this context, the present analysis should be understood as a pragmatic application of generic ITC guidance to an ultra-rare disease setting, with the aim of assessing whether foundational assumptions for credible indirect comparison can be reasonably satisfied with currently available evidence.

## Materials & methods

### Feasibility assessment of conducting ITCs between iptacopan & pegcetacoplan based on APPEAR-C3G & VALIANT RCTs

In this methodological appraisal, NICE DSU and ISPOR ITC guidance documents [1–4] are leveraged to explore the complexity of conducting ITCs in ultra-rare disease areas and to highlight the dearth of valid ITCs in an ultra-disease area such as C3G. We evaluated whether core assumptions for valid anchored and unanchored ITCs could be satisfied using trial publications and registries for APPEAR-C3G [15–17] and VALIANT [18–21], interpreted against generic NICE DSU TSD-18 and CHTE2020, and ISPOR good-practice guidance [1–4].

We compared key elements across trials, including eligibility criteria (age, disease subtype, transplant status), design features, and primary endpoint definitions. As per NICE DSU TSD-18, CHTE2020 and ISPOR good-practice guidance, the criteria for assessment were major population nonoverlap (e.g., age group, transplant status, disease type (IC-MPGN or C3G) and end point heterogeneity (first morning void [FMV] vs 24 h urine protein-creatinine ratio [UPCR]; differing eGFR estimands) [1–4].

We also assessed placebo response comparability as a proxy for cross-trial differences after alignment, examined similarity of placebo responses (UPCR, eGFR) and the consistency of measurement frameworks (FMV vs 24 h; least squares [LS] mean vs slope/absolute mean differences [AMD]) [1–4,15–20,22]. We then assessed anchored assumptions, (i.e., the plausibility of preserving randomization given differences in placebo response); when placebo responses diverge, the anchor weakens and Bucher and matching-adjusted indirect comparison (MAIC) estimates are at risk of bias [1–4].

### Systematic literature review to identify published ITCs in C3G

#### Search strategy

A systematic literature review (SLR) search was conducted in Embase (up to 4 March 2026), MEDLINE (up to 5 March 2026), and Cochrane Database of Systematic Reviews via Ovid (up to 4 March 2026) to identify abstracts, posters and publications assessing the indirect effects between iptacopan and pegcetacoplan treatments in C3G. Time restrictions were not included in the search, other than a 3-year retention filter for abstracts identified in Embase (2023-current). Using population-, condition- and ITC-specific search terms, a search strategy was developed by an information specialist (Supplementary Tables A1 & A2) [23]. This was supplemented by searches of grey literature, and manual searches of conference proceedings from the European Renal Association (ERA), the American Society of Nephrology (ASN), the World Congress of Nephrology (WCN) and the reference lists of included studies, for the last 3 years (2023–2025). Searches of conference proceedings used disease search terms (C3G; C3 Glomerulopathy; C3GN; C3 Glomerulonephritis; Dense Deposit Disease; DDD; Membranoproliferative Glomerulonephritis; MPGN; Mesangiocapillary Glomerulonephritis; MCGN) and study design search terms (ITC; NMA; Indirect treatment comparison; Network meta-analysis). A protocol was not prepared or registered.

#### Eligibility criteria

Eligible studies included ITCs, such as MAIC, simulated treatment comparison (STC), NMA and multilevel network meta-regression (ML-NMR). We also included studies with patients diagnosed with C3G, and those which investigated iptacopan or pegcetacoplan. Studies were required to report at least one outcome related to efficacy (including but not limited to changes in UPCR, changes in eGFR, eGFR stabilization, C3G histological score for disease, changes in hematuria, mortality and time to end-stage kidney disease), safety (adverse events, severe infections) and treatment discontinuation).

#### Study selection

Two reviewers independently screened titles, abstracts and full texts using the predefined eligibility criteria, with disagreements resolved by a third reviewer. All ITCs that met inclusion criteria were extracted and appraised in this manuscript.

#### Data extraction

To support the assessment of ITC feasibility, relevant data were extracted from each trial included in the SLR-identified ITC. These data included:

- Patient eligibility criteria and population scope (e.g., age bands; native vs post-transplant C3G; inclusion/exclusion of IC-MPGN). Data were cross-checked with peer-reviewed publications and registries [15–20].
- Baseline characteristics (e.g., age, UPCR and eGFR by randomized arm) as reported in trial publications/registries [15–20].
- Endpoint definitions and timing, including proteinuria assessments (FMV vs 24 h UPCR) and kidney function reporting [15–20].

Independent reviewers resolved discrepancies by consensus and verification against source documents.

#### Methodological quality assessment

The SLR designed in this study was intended to identify ITCs conducted between the Phase III trials, APPEAR-C3G and VALIANT, rather than to synthesize a broader body of evidence. As such, a quality assessment was not conducted.

#### Critical appraisal of comparative ITCs identified in SLR

ITCs identified by the SLR were critically appraised with respect to NICE DSU and ISPOR guidance [1–4], focusing on:

- Methodological rigor:
  ○ We assessed the methodological rigor of the identified ITC by examining whether the selected ITC approach was appropriate. This included evaluating the comparability of included studies and the appropriateness of ITC methods. We reviewed whether covariate selection, matching eligibility criteria (e.g., rules to match VALIANT to APPEAR-C3G eligibility), weighting and other adjustment strategies were applied appropriately.
- Transparency and interpretability:
  ○ We assessed the transparency and interpretability of the identified ITC by reviewing the clarity and completeness of methodological reporting. Specifically, we evaluated the documentation of study selection criteria, baseline characteristics, and outcome definitions. We examined whether sufficient detail on diagnostics for population-adjusted analyses was provided (e.g., ESS, weight distributions, covariate balance), and whether post-alignment sample sizes and characteristics were reported. We assessed whether the identified ITC adhered to NICE DSU and ISPOR reporting expectations.
- Credibility:
  ○ We evaluated the extent to which the identified ITC acknowledged assumptions, uncertainty and whether conclusions were presented cautiously and without overinterpretation. For example, whether the results were communicated in a balanced manner given the findings and methodological limitations.

#### Ethics approval & consent to participate

This study used publicly available clinical trial publications and ClinicalTrials.gov registry data for APPEAR-C3G [15–17] and VALIANT [18–21] and publicly available ITC publications identified by a SLR, so no additional ethics approval or informed consent was required. Additionally, no individual patient data were analyzed.

## Results

### Feasibility assessment of conducting ITCs between iptacopan & pegcetacoplan based on APPEAR-C3G & VALIANT RCTs

#### Differences in baseline characteristics between APPEAR-C3G & VALIANT do not adhere to similarity assumptions

Across the two Phase III trials compared via ITC, major differences in eligible populations and patient populations were identified (Table 1 & Supplementary Table A4). APPEAR-C3G randomized adult native C3G patients in its primary analysis population and required reduced serum C3 at baseline; it excluded transplant and IC-MPGN patients. APPEAR-C3G used 24 h UPCR consistently throughout the double-blind period and into the open-label extension [15–17]. Baseline 24 h UPCR was collected within 7 days of randomization, with background medications required to be stable for 90 days prior to baseline.

**Table 1.** Comparison of key patient and study characteristics between APPEAR-C3G (iptacopan) and VALIANT (pegcetacoplan).

Characteristic	APPEAR-C3G (iptacopan)	VALIANT (pegcetacoplan)
Study overview		
Study design	• Phase III, multicenter, randomized, double-blind, parallel group, placebo-controlled study	• Phase III, multicenter, randomized, double-blind, placebo-controlled study
Comparators	• Placebo	• Placebo
Dosing	• Standard dose	• Adults: standard dose • Adolescents: **weight-adjusted doses for those ≤50 kg**
Key inclusion criteria		
Patient characteristics		
Age	• Adults (≥18–60 years)	• Adults (≥18 years); OR • **Adolescents (≥12–17 years)**^†^
Diagnosis	• Diagnosis of C3G^‡^	• Diagnosis of C3G^§^, OR • **Diagnosis of IC-MPGN**^§^
Prior treatment	• Required to have been receiving the maximally recommended or tolerated dose of an ACEi or ARB for ≥90 days prior to randomization • Other antiproteinuric medications^¶^ should be stable for ≥90 days prior to randomization	• Required to have been receiving stable/optimized ACEi or ARB, for ≥84 days prior to randomization • Other antiproteinuric medications^#^ should be stable for ≥84 days prior to randomization, and ≤20 mg/day (or equivalent dosage of a corticosteroid other than prednisone)
Measurements of disease activity		
C3c staining	• Not specified	• Adults: **≥2+ staining** • Adolescents: **≥2+ staining**^§^
Serum C3 levels	• Reduced serum C3 (≤0.85 LLN during screening)	• Adults: **no restrictions** • Adolescents: Reduced serum C3 below the LLN^††^
Proteinuria	• Not specified	**• ≥1 g/day on a screening 24 h urine collection**
UPCR	• UPCR ≥1.0 g/g (FMV) on days -75 and -15	**• UPCR ≥1.0 g/g (24 h urine) on 2 measurements prior to screening**
eGFR	• eGFR ≥30 ml/min/1.73 m^2^^‡‡^	• eGFR ≥30 mL/min/1.73 m^2^^§§^
Histology	• Kidney biopsy showing IF/TA of >50%	Glomerulosclerosis or tubular atrophy tubulointerstitial scarring on the baseline biopsy: • Adults: **≤50%** • Adolescents: **≤50% for those providing a baseline biopsy**
Key exclusion criteria		
Transplant history	• Participants who have received any cell or organ transplantation, including a kidney transplantation were excluded	• **No exclusion criteria specified**
Treatment history	• The use of inhibitors of complement factors (e.g., Factor B, Factor D, C3 inhibitors, anti C5 antibodies, C5a receptor antagonists) within 6 months prior to the screening visit.	• Use of rituximab, belimumab, or any approved or investigational anticomplement therapy other than pegcetacoplan within 5 half-lives of that product prior to the screening period.
Weight	• No exclusion criteria specified	• **Participants with a body weight >100 kg at screening were excluded**
Disease description	• C3G secondary to monoclonal gammopathy, or acute post-infectious glomerulonephritis	• C3G/IC-MPGN secondary to another condition (e.g., infection, malignancy, monoclonal gammopathy, a systemic autoimmune disease such as systemic lupus erythematosus, chronic antibody-mediated rejection or a medication), in the opinion of the investigator.

Orange cells with bolded text indicates differences between trials that should be considered when evaluating the feasibility of an ITC.

† Weighing at least 30 kg.

‡ As confirmed by renal biopsy within 12 months prior to enrollment in adults and within 3 years in adolescents.

§ Baseline biopsies were not required for adolescent participants if they had an adequate previous renal biopsy to establish the diagnosis, even if the previous biopsy was done more than 28 weeks before randomization.

¶ Mycophenolic acid, corticosteroids, SGLT2 inhibitors and mineralocorticoid receptor antagonists.

# Steroids, mycophenolate mofetil, SGLT2 inhibitors and/or other allowed immunosuppressants that the participant is receiving for treatment of C3G or IC-MPGN.

†† In adolescents not providing a baseline renal biopsy, serum C3 below the LLN during screening would satisfy the ‘evidence of active renal disease’ inclusion criteria.

‡‡ Estimated GFR (using the CKD-EPI formula for ages ≥18 years or measured GFR ≥30 ml/min/1.73 m^2^ at screening and day -15.

§§ Calculated by the Chronic Kidney Disease-Epidemiology Collaboration creatinine equation for adults. For participants initially screened as adolescents, eGFR was calculated using the Schwartz formula throughout the study, even if they turned 18 years old during the trial.

ACEi: Angiotensin-converting enzyme inhibitor; ARB: Angiotensin receptor blocker; C3G: Complement 3 glomerulopathy; eGFR: Estimated glomerular filtration rate; FMV: First morning void; IC-MPGN: Immune-complex membranoproliferative glomerulonephritis; IF: Interstitial fibrosis; LLN: Lower limit of normal; MMF: Mycophenolate mofetil; SGLT2i: Sodium-glucose cotransporter-2 inhibitor; TA: Tubular atrophy; UPCR: Urine protein-to-creatinine ratio.

Data taken from [15–20].

In contrast, VALIANT randomized both adolescent (12 to 17 years of age) and adult patients (≥18 years of age), included native C3G, post-transplant recurrent C3G, and primary IC-MPGN, and assessed the primary proteinuria end point using equal-weighted average of FMV UPCR over weeks 24–26 (with 24 h urine obtained at screening) [18–21]. VALIANT compared week 24–26 FMV UPCR with FMV measures collected during screening (over ∼8 weeks), so baseline UPCR could reflect values obtained some time before randomization. Because medications which can reduce UPCR were not required to be stable during the longer screening period in VALIANT, baseline UPCR in VALIANT may be inflated; exclusion of patients with >50% screening improvement only partially mitigates this between-trial difference.

Between-trial differences were apparent in the baseline characteristics of each trial. While an adolescent cohort has been enrolled in APPEAR-C3G, results of this cohort are not yet available (Table 1). Therefore, an ITC comparing an adult only cohort from APPEAR-C3G to a combined adolescent and adult cohort from the VALIANT trial (0% vs 44%) would not satisfy the similarity assumption required for anchored ITCs, thus undermining credibility of results [16].

APPEAR-C3G [15–17] included fewer female participants than VALIANT (36.5% vs 56%) (Table 2) [18–21]. APPEAR-C3G excluded participants with history of transplant, whereas VALIANT included transplant patients (0% vs 7%). APPEAR-C3G and VALIANT had notably different median (range) serum C3 levels at baseline (295.5 [20–950] vs 600 [100–1600]). These between-trial differences in baseline characteristics do not satisfy the exchangeability and end point harmonization expectations for anchored ITCs, articulated by generic NICE DSU TSD-18 and ISPOR guidelines. Therefore, anchored ITCs based upon these data may be biased and results should be interpreted with caution [1–4].

**Table 2.** Comparison of key baseline patient characteristics and outcomes between APPEAR-C3G (iptacopan) and VALIANT (pegcetacoplan).

Characteristic	APPEAR-C3G (iptacopan)		VALIANT (pegcetacoplan)^†^	
Demographic characteristics				
Number of total patients, N	• 74		• **124**	
Female n (%)	• 27 (36.5)		• **70 (56)**	
Population subgroups				
History of transplant, n (%)	• 0 (0)		• **9 (7)**	
Age group, n (%)	• Adolescent (12–17 years): 0 (0) • Adult (≥18 yr): 74 (100)		• **Adolescent (12–17 years): 55 (44)** • Adult (≥18 years): 69 (56)	
Underlying disease, n (%)	• C3G: 74 (100) • IC-MPGN: 0 (0)		• C3G: 96 (77) • **IC-MPGN: 28 (23)**	
Measurements of disease activity at baseline				
Median (range) C3, mg/l	• 295.5 (20–950)		• **600.0 (100–1600)**	
Mean (SD) UPCR, g/g	• 3.40 (2.066)		• 2.873 (2.234)^‡^	
Median (range) eGFR, ml/min/1.73 m^2^	• 104.26 (28.1–135.9)		• 85.5 (24–161)	
Key outcome results				
Treatment groups:	Iptacopan	Placebo	Pegcetacoplan	Placebo
Mean reduction in log-transformed UPCR from baseline at 6 months	• -30.2% (-42.8, -14.8)	• 7.6% (-11.9, 31.3)	• **-67.2% (-74.9, -57.2)**	• **2.9% (-8.6, 15.9)**
Proportion of patients who achieved UPCR <1 g/g at 6 months	• 10.5%	• 5.6%	• 50.8%	• 19.7%
Proportion of patients who achieved ≥50% UPCR reduction from baseline at 6 months	• 30%	• 6%	• 60%	• 5%
Change in eGFR (ml/min per 1·73 m^2^) from baseline to 6 months (95% CI)	• 1.30 (-2.14, 4.73)	• -0.86 (-4.36, 2.64)	• **-1.5** (-5.9, 2.9)	• **-7.8** (-11.6, -4.0)
Proportion of patients who achieved the composite renal end point^§^ at 6 months	• 30%	• 6%	• 49%	• 3%

Orange cells with bolded text indicates differences between trials that should be considered when evaluating the feasibility of an ITC.

† Limited subgroup data has been published form the VALIANT trial. Therefore, results included in this table are derived from all study participants (ie, including participants with prior transplants, IC-MPGN, and who are adolescents).

‡ Baseline triplicate first-morning spot urine protein-to-creatinine ratio, converted to g/g.

§ Defined as a ≥50% reduction in UPCR from baseline and ≤15% reduction in eGFR from baseline.

C3G: C3 glomerulopathy; eGFR: Estimated glomerular filtration rate; IC-MPGN: Immune-complex membranoproliferative glomerulonephritis; UPCR: Urine protein-to-creatinine ratio.

Data taken from [15–20].

#### Noncomparability of placebo anchors

Large differences were noted between placebo arms for all outcomes in APPEAR-C3G and VALIANT (Table 2), with pronounced differences for mean reduction in log-transformed UPCR (7.6% vs 2.9%) and change in eGFR from baseline at 6 months (-0.86 vs -7.8 ml/min/1.73 m^2^), reflecting dissimilar enrollment criteria, disease populations and the limited sample sizes between trials [15–20]. Moreover, placebo outcomes are not directly comparable due to FMV versus 24 h UPCR and different eGFR estimates (LS mean changes vs adjusted mean differences) [15–20].

#### ITC feasibility summary

In consideration of population nonoverlap (age range, transplant status, disease status, diagnosis), baseline imbalances, endpoint heterogeneity (FMV vs 24 h; LS mean vs AMD/slope) and noncomparable placebo anchors, valid, anchored ITCs comparing iptacopan and pegcetacoplan (using currently available APPEAR-C3G and VALIANT trial data) are not feasible without extensive, transparent harmonization and population adjustment (including full diagnostics and ESS reporting) as per NICE DSU and ISPOR guidance [1–4,15–20].

#### Systematic literature review of published ITCs in C3G

The SLR identified 12 unique records through databases and one record through manual searches of conference proceedings. Only one record described an ITC that met the SLR eligibility criteria, which was a conference poster by Dixon *et al.* [24] comparing iptacopan and pegcetacoplan in C3G. Dixon *et al.* compared iptacopan and pegcetacoplan in C3G based on data from APPEAR-C3G [15–17] and VALIANT [18–20].

Other records were excluded for not including a C3G population (n = 8), or for not presenting findings from ITC or NMA analyses (n = 4). A list of excluded studies is provided in Supplementary Table A3, and the related PRISMA flow diagram is provided in Supplementary Figure A1 [23]. No additional ITCs or NMAs were found across databases or conferences.

An additional poster presentation by Dixon *et al.* [25], was published following the initial SLR and was identified for inclusion through a supplemental search. Finally, during the peer-review process for this manuscript, the associated, peer reviewed full text ITC report by Dixon *et al.* [26] became available, which included the full analysis and methods of the previously identified poster records [24,25]. As the newly identified manuscript contained additional details, it was incorporated into our assessment to ensure that all relevant methodological details and results were considered [26].

### Critical appraisal of comparative ITCs identified by SLR

#### Methodological rigor

**Figure 1.**
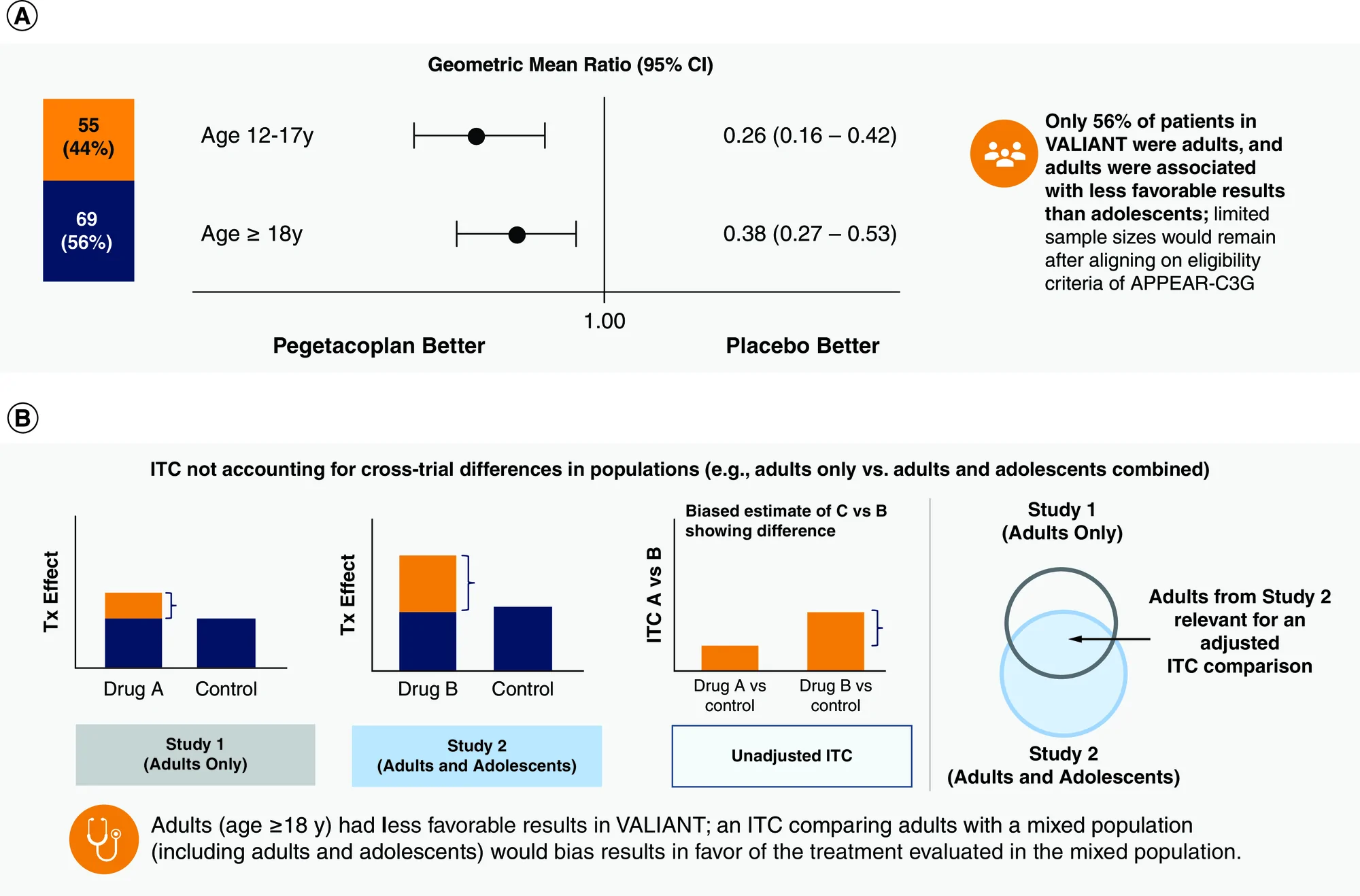
Cross-trial differences are essential for unbiased ITC estimates. **(A)** Participants in the VALIANT trial include subgroup populations with more favorable results (e.g., adolescents) [18–20]. **(B)** Given that VALIANT data represent a combined adult and adolescent population, whereas the available APPEAR-C3G data reflect adults only, failing to account for these differences could bias results in the ITCs (left). It is considered best practice in ITCs to assess and adjust for cross-trial differences in populations. This ensures that comparisons are made between appropriately aligned subgroups, such as restricting analyses to adults from Study 2 when comparing against summary-level data from an all-adult Study 1, to improve the validity and interpretability of treatment effect estimates (right). IC-MPGN: Immune Complex-Mediated Membranoproliferative Glomerulonephritis; ITC: indirect treatment comparison; Tx: treatment.

**Figure 2.**
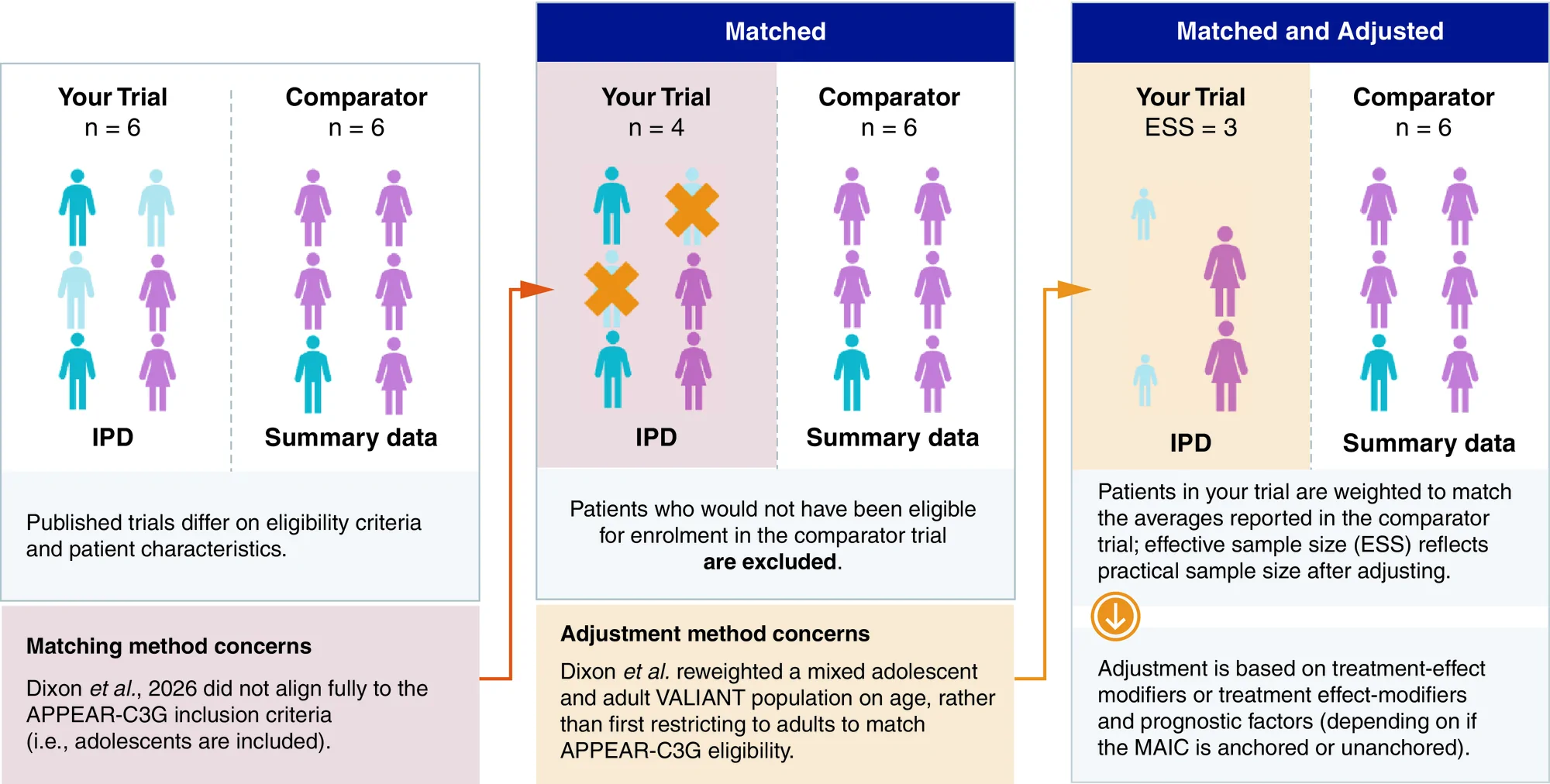
Matching adjustment indirect comparison methodology and reporting requirements. Applying the APPEAR-C3G eligibility criteria to the VALIANT population substantially reduces the number of adult native C3G patients available for analysis. A limited sample size heightens the risk of residual confounding, unstable weighting, and low ESS in the MAIC. Dixon *et al.*, 2026 [26] retained adolescents in the VALIANT sample to preserve ESS, rather than restricting to adults as APPEAR-C3G eligibility requires. In doing so, the initial MAIC matching step was not fully completed, undermining the reliability of downstream adjustments and the interpretability of the final MAIC results. ESS: Effective sample size; IPD: Individual patient data; MAIC: Matching-adjusted indirect comparison. Data taken from [28,29].

The choice of anchored MAIC methodology by Dixon *et al.* [26] was appropriate given the cross-trial differences, and the common comparator between the trials. Given that Dixon *et al.* [26] had access to VALIANT individual patient data (IPD) and published aggregate data from APPEAR-C3G, restricting the VALIANT population to patients meeting APPEAR-C3G eligibility and weighting to adjust for residual cross-trial differences was a reasonable approach. In practice, Dixon *et al.* applied APPEAR-C3G's eligibility criteria to the VALIANT population only using four criteria (exclusion of patients with primary IC-MPGN, prior renal transplant, prednisone >7.5 mg/day [or equivalent] within 90 days of study drug, or baseline serum C3 ≥77 mg/dL) but did not exclude on age, instead, attempting to reconcile the age difference by using age as the sole weighting variable in the MAIC. As shown in Figures 1 & 2, VALIANT's broader enrollment criteria (adolescents; transplant; IC-MPGN) creates limited overlap with the APPEAR-C3G adult native population. Aligning the VALIANT population to APPEAR-C3G via the VALIANT eligibility criteria would necessarily shrink the analysis set to a small subset of adult native C3G, thus increasing the likelihood of residual confounding, unstable weights and low ESS in the MAIC (Figure 2) [27]. Retaining adolescents across Bucher and MAIC analyses preserved ESS but reduced comparability, as the VALIANT sample extended beyond the enrolled APPEAR-C3G population.

The poster publications by Dixon *et al.* [24,25] did not sufficiently outline the rationale of covariate selection for adjustment in the ITC (e.g., systematic review of prognostic factors/treatment effect modifiers [TEMs], or consultation with clinical experts), which may bias results through omitted-variable bias. The full Dixon *et al.* manuscript publication [26] confirms that only baseline age was used as a reweighting variable in the anchored MAIC, with the authors stating that age alone reflected the difference in available data between the trials (VALIANT enrolled adolescents and adults; APPEAR-C3G enrolled adults only) and preserved sample size. However, no formal effect-modifier identification process (e.g., systematic prognostic factor review or clinical-expert consultation) is reported (Figure 2). Not surprisingly, baseline data reported in the full manuscript confirm that, after reweighting on baseline age alone, several baseline characteristics remain significantly imbalanced across trial arms, including sex (35.9% male in pegcetacoplan vs 71.1% male in iptacopan; p = 0.007), disease subtype (DDD prevalence 6.2% vs 23.7%; p = 0.032), and hypertension among placebo arms (9.6% vs 50.0%; p < 0.001), limiting the interpretation of ITCs. Without a clear, pre-specified covariate selection methodology and fully transparent diagnostics of appropriately adjusted ESS or a plot of patient weights, the technical validity of the adjustments cannot be fully verified.

Dixon *et al.* [26] also presented findings from an unanchored MAIC. It is well established that unanchored MAICs are less methodologically rigorous than anchored MAICs given the additional requirements to adjust for both treatment effect modifiers and prognostic factors [2].

Despite this additional methodological requirement outlined in NICE TSD [2], the full publication reported by Dixon *et al.* [26] used age as the sole matching variable in both the anchored and unanchored MAICs. The persistent baseline imbalances noted above reinforce that age-only adjustment was insufficient to ensure comparability, and that these results should be interpreted with caution.

#### Transparency & interpretability

Dixon *et al.* [26] adequately reported Bucher results for the full trial-level populations, but the abstract and associated poster did not disclose the post-alignment ESS and characteristics from MAICs, preventing verification that key effect modifiers were adequately balanced, an important reporting element emphasized by both NICE DSU and ISPOR guidance [1–4,24,27]. Furthermore, the posters did not report weight diagnostics or the ESS after weighting in MAICs [24]. Despite the fact that results were presented in a poster publication, which was therefore space-constrained, when given the opportunity to disclose more details on patient characteristics in the format of a manuscript, the ESS values reported in the manuscript include adolescents in the calculation, rather than being restricted to the adult-only overlap with APPEAR-C3G [26].

The full publication by Dixon *et al.* [26] cited ESS values that included adolescents in the Bucher and in the MAICs, instead of restricting to the appropriate adult population overlap between VALIANT and APPEAR-C3G, undermining the credibility of the comparison. The post-harmonization VALIANT analysis set is n = 49 (pegcetacoplan = 26, placebo = 23), with reported post-weighting ESS of 22 (84.6%) for the pegcetacoplan arm and 7 (30.4%) for the placebo arm. These estimates are likely inflated, as the authors did not align eligibility criteria on age as they included adolescents rather than restricting the population to adults [26]. Nevertheless, the inflated ESS of 22 and 7, are well below the level at which MAIC estimates can be considered methodologically stable [27], reinforcing the credibility concerns already outlined above.

#### Credibility

Given the limited overlap between APPEAR-C3G [15–17] and VALIANT [18–20], the aligned subset from VALIANT (adult native C3G only) is likely small, which renders the analysis susceptible to extreme weights, low ESS (published in the Dixon *et al.*, 2026 full-text publication [26] as ESS = 22 for the pegcetacoplan arm and ESS = 7 for the placebo arm), and unstable ITC estimates (Figures 1 & 2). The inclusion of adolescents from the VALIANT trial means there is a strong possibility that results are biased by a few heavily weighted individuals [26]. While the publications by Dixon *et al.* acknowledge methodological limitations and potential risk of bias in the ITC results, the manuscript's conclusions and the guidance provided to clinicians and payers evaluating new therapies for C3G do not appear to fully reflect this degree of uncertainty.

## Discussion

### Summary of main findings

Underscoring the very limited comparative evidence base currently available in the ultra-rare disease C3G, the SLR identified just one ITC meeting eligibility criteria, published as a conference poster by Dixon *et al.* [24], comparing iptacopan and pegcetacoplan. During the peer review process of this manuscript, Dixon *et al.* published an encore at WCN 2026 [25] as well as the associated, peer reviewed full text ITC manuscript [26]. Using the ITC guidelines set out by the NICE DSU and ISPOR task force [1–4], we conducted an ITC feasibility assessment that focused upon the underlying APPEAR-C3G [15–17] and VALIANT [18–20] trials and then critically appraised all materials related to the analyses presented by Dixon *et al.* [24–26]. Our appraisal found that anchored ITCs between iptacopan (APPEAR-C3G) and pegcetacoplan (VALIANT), using currently available data, are not feasible without substantial methodological compromises that do not satisfy ITC guidelines set out by the NICE DSU and ISPOR task force [1–4]. As detailed in Table 1, the two currently available C3G Phase III trials differ on essential baseline characteristics that underpin ITC validity, including population scope (age range, transplant status, disease status, diagnosis), biomarker entry criteria (serum C3 requirement in APPEAR-C3G, none in VALIANT), and end point measurements and estimands (e.g. different outcome definitions and measurements in UPCR and eGFR) [15–20,24]. C3G experts have further noted substantial variability in eGFR changes from baseline among placebo participants across C3G trials [30]. Without these crucial details, the ITC publications by Dixon *et al.* [24–26] are subject to interpretability and transparency issues, thus undermining credibility of conclusions.

Once published, Dixon *et al.* [26] disclosed several key methodological details that had not been reported in the earlier conference materials, including the post-alignment analysis set, baseline characteristics after weighting, and the ESS values for the matched populations. While the publication improves transparency, it also introduces an unanchored MAIC, which raises additional credibility concerns because such analyses require stronger assumptions that all relevant prognostic variables and effect modifiers have been accounted for [26].

MAICs are known to incur substantial ESS losses when overlap between trial populations is limited. In a review of MAICs in NICE technology appraisals, Phillippo *et al.* reported, among the 9 appraisals reporting ESS, a median post-weighting ESS of 80 (range: 4.0 to 335.5), with a median reduction from the original sample size of 74.2% (range: 7.9% to 94.1%). It is worth noting that 83% of assessed NICE technology appraisals were in oncology indications, and suitable ESS proxies for ultra-rare renal diseases are not currently available [31]. The reported anchored MAIC ESS values in the analyses reported by Dixon *et al.* (22 for the pegcetacoplan arm and 7 for the placebo arm) sit well below the median of this NICE distribution, with the placebo ESS of 7 approaching the minimum (4.0) reported across NICE submissions, reinforcing concerns about instability and potential bias [26,31]. Even with the inclusion of adolescents, the reported ESS values fall within the range at which MAIC estimates have been shown to be unstable, highly sensitive to extreme weights, and increasingly prone to bias in rare-disease settings, as summarized by Parkitny *et al.* [27]. Parkitny *et al.* identify ESS below approximately 30 as a threshold below which MAIC estimates are prone to bias and note that, under such conditions, the treatment effect may effectively be driven by the outcomes of only one or two heavily-weighted individuals [27].

In the context of VALIANT's broader enrollment criteria, any alignment to APPEAR-C3G's adult-native population would necessarily yield a small subset (Figure 2), amplifying risks of unstable weighting and ESS collapse (per NICE DSU recommendations), potentially biasing the results [1–4,24,27]. These findings highlight both the challenges of applying existing ITC frameworks in ultra-rare diseases and the need for ongoing methodological development and clearer guidance on indirect comparisons in rare disease settings.

Currently available ITCs in C3G should therefore be interpreted with caution. Substantial cross-trial heterogeneity between APPEAR-C3G [15–17] and VALIANT [18–20], together with the very small patient subsets remaining after alignment on selected eligibility criteria, suggest that the assumptions underlying standard ITC and population-adjusted methods are not satisfied in this rare disease setting. This concern is consistent with broader methodological evidence showing that published anchored MAICs often produce results more favorable to the IPD-informed treatment than related standard anchored [27,32]. Moreover, Cassidy *et al.* emphasizes that MAICs should be reported as transparently as possible, given the subjective nature of covariate selection and the need for HTA assessors to evaluate the credibility of the adjustment [32].

### Implications for clinical, market access & HTA decision-making

Given these methodological considerations, any comparative efficacy claims between iptacopan and pegcetacoplan in C3G, using currently available data and derived from anchored or unanchored ITCs, should be interpreted with caution. Decision-makers should instead prioritize trial-specific interpretations (i.e., within-trial randomized comparisons as reported in peer-reviewed sources) and request full transparency for any future population-adjusted ITC analysis, including sample sizes following population alignment, process of covariate selection and balance after weighting, weight distributions and full disclosure of appropriate ESS [1–4]. In ultra-rare diseases like C3G, where sample sizes are small and studies vary in design and end point reporting, comparisons of treatment effects should be interpreted carefully [1–4].

### Practical recommendations for future ITCs in C3G

#### Present transparent population-adjustment methods & diagnostics

When employing MAIC, ML-NMR or STC methods, disclose the aligned analysis set, identification methods and final listing of covariate selection (effect modifiers/prognostic factors), weight diagnostics and ESS after weighting, as recommended by NICE DSU and ISPOR [1–4]. Inclusio

n of this information provides clarity for readers who must assess such analyses and ultimately establish a degree of confidence in the findings presented.

#### Follow publication standards

Adhere to peer-reviewed reporting requirements and journals' methodological checklists to ensure that comparative evidence is reproducible, credible, and HTA-ready [1–4].

#### Transparent data on sub-populations

Provide clear, disaggregated subgroup summaries, including baseline characteristics, sample sizes and outcome data for relevant clinical subpopulations to allow others to reproduce or re-evaluate ITCs using consistent definitions.

Recent methodological developments suggest that, where feasible, model-based approaches such as ML-NMR represent a more appropriate option than anchored MAIC for population-adjusted comparisons under current generic NICE DSU guidance [1,33,34]. More broadly, generic NICE and ISPOR frameworks allow consideration of alternative approaches, including unanchored MAIC, Bayesian NMA with informative priors, ML-NMR or qualitative structured cross-trial synthesis with transparent caveats in data-limited settings. Nevertheless, in ultra-rare disease contexts such as C3G, very small sample sizes, limited covariate overlap and incomplete understanding of prognostic and effect-modifying factors impose fundamental constraints across all population-adjusted methods. While these methods may reduce the visibility of low ESS by relying on outcome modeling rather than reweighting, they remain vulnerable to extrapolation and strong model dependence when data are sparse, underscoring the need for caution in interpreting results regardless of the adjustment framework used.

### Strengths & limitations

Strengths of this investigation include use of peer-reviewed publications and ClinicalTrials.gov records for APPEAR-C3G [15–17] and VALIANT [18–20], the systematic mapping of trial differences into an ITC feasibility framework grounded in NICE DSU and ISPOR guidance [1–4], and provision of high-quality tables and figures to visualize the practical barriers to valid ITCs.

Several limitations should also be acknowledged. Our appraisal relied on publicly available poster and full-text sources without access to individual patient data [24–26]. Although the full-text publication disclosed additional methodological detail, the complete distribution of patient weights remains unavailable for verification, and therefore, we did not conduct *de novo* population-adjusted ITCs [26]. The SLR was not prospectively registered (e.g., via PROSPERO), although the search strategy, eligibility criteria and analytical approach were defined a priori and applied consistently. Finally, as no validated risk-of-bias tool currently exists for population-adjusted indirect comparisons, no formal credibility assessment was undertaken. Instead, our appraisal followed established best-practice guidance (NICE DSU and ISPOR) and PRISMA reporting standards, and explicitly examined sources of MAIC bias, including covariate and effect-modifier selection, reliance on unanchored comparison and loss of ESS after weighting.

## Conclusion

Currently available ITCs in C3G should be interpreted with caution. Substantial cross-trial heterogeneity between APPEAR-C3G [15–17] and VALIANT [18–21], along with the very small, highly restricted patient subsets remaining after matching on eligibility criteria, indicate that the core assumptions specified in existing ITC frameworks are difficult to satisfy in this ultra-rare disease context using currently available data. The appraisal of ITC publications from Dixon *et al.* [24–26] performed here highlights credibility concerns. Although the full manuscript improves transparency relative to earlier conference materials, key limitations remain, including the small ESS in the anchored MAIC and the stronger assumptions required for the unanchored MAIC. Accordingly, payers, healthcare decision-makers, and HTA bodies should require clear population alignment, justified covariate selection, demonstrated covariate balance and appropriate weight diagnostics before relying on comparative claims between iptacopan and pegcetacoplan in C3G.

## Summary points

- This manuscript presents a methodological appraisal of indirect treatment comparisons (ITCs) in C3 glomerulopathy (C3G) using publicly available trial data and published analyses.
- C3G is an ultra-rare, complement mediated kidney disease, posing inherent challenges for evidence generation and comparative effectiveness assessment.
- The Phase III trials, APPEAR C3G (iptacopan) and VALIANT (pegcetacoplan), trials differ substantially in eligibility criteria, patient populations, and study design.
- Important cross trial differences were identified in age distribution, disease subtype, transplant status, biomarker requirements and baseline characteristics.
- Endpoint definitions and measurement approaches, particularly for proteinuria and kidney function, were not harmonized across trials.
- Placebo responses differed meaningfully between trials, weakening the comparability of common anchors required for valid ITCs.
- A systematic literature review (SLR) identified only one published record of an ITC study, reflecting the extremely limited comparative evidence base in C3G. Subsequently, an additional poster and full-text manuscript describing the same ITC study were published and incorporated into this appraisal.
- The identified ITC employed a matching adjusted indirect comparison but, despite subsequent disclosure of post-alignment characteristics and effective sample sizes (ESS) in the full publication, retains methodological limitations including reweighting on a single variable (baseline age), residual baseline imbalances after weighting, an unreported weight distribution and a very low ESS (pegcetacoplan n = 22 and placebo n = 7).
- Limited population overlap implies substantial ESS reductions, increasing the risk of unstable weighting and biased estimates.
- These findings underscore the need for cautious interpretation of existing ITCs and for greater methodological transparency in rare disease settings.

## Supplementary Material




