# Supplementary material for: Methodological pitfalls in indirect treatment comparisons: insights from a recent systematic review and analysis for C3 glomerulopathy
Source: J Comp Eff Res. 2026 Aug 9;15(9):e260054. doi: 10.57264/cer-2026-0054 (PMC13556872; doi:10.57264/cer-2026-0054)
Supplement: Supplementary file 1 [file cer-15-260054-s1.docx]

# Appendix

**Appendix Table 1: Patient, Intervention, Comparison, Outcome, and Study Design (PICOS) Criteria**

| **Inclusion criteria** | **Exclusion criteria** |
| --- | --- |
| Patient population:  **Patients diagnosed with C3G** | Any other patient population |
| Intervention**:**  **Any intervention for C3G patients** | NA |
| Comparator:  **Any intervention for C3G (including best supportive care) or placebo** | NA |
| Outcomes* (including but not limited to):  **Efficacy (e.g., changes in urine protein to creatinine ratio, changes in proteinuria (mean change and proportion of patients to reach proteinuria below 1 g/day from baseline - partial and complete remission), changes in eGFR, eGFR stabilization, C3G histological score for disease, changes in hematuria, mortality, time to ESKD etc.), safety (adverse events [AEs], severe infections) and treatment discontinuation** | Studies not reporting relevant outcomes of interest |
| Study design:  **Indirect-treatment comparisons**  **Network meta-analysis** | Case reports, case series, editorials, letters, protocols, opinion papers, commentaries, news, narrative and systematic review, non-human and in vitro studies, observational/real-world studies, clinical trials etc |
| Language: **No language restriction** | NA |
| Publication date: **2006 to 2026** | NA |
| Country: **No country restriction** | NA |

**Appendix Table 2: SLR search strategy conducted on March 6, 2026.**

| **#** | **Searches** | **Results** |
| --- | --- | --- |
| 1 | (Glomerulonephritis, Membranoproliferative/ and (((c3 or c-3 or serum-c3 or globulin-beta1-c or (globulin adj2 beta adj2 ("1c" or "1 c")) or ((complement? or component? or precursor?) adj1 "3")) adj4 glomerulo$) or ((C3GN or C3G or MPGN$3 or DDDMPGN$3 or 80295-41-6) and glomerulo$)).mp.) or (exp complement c3/ and (((C3GN or C3G or MPGN$3 or DDDMPGN$3 or 80295-41-6) and glomerulo$) or (dense adj3 deposit adj3 disease?) or ((membranoproliferat$ or membrano-proliferat$ or mesangiocapillar$ or mesangio-capillar$) adj4 (glomerulonephritis$ or glomerulo-nephriti$) adj4 (type-ii or type-2 or subendothelial$ or sub-endothelial$)) or (MPGN adj4 (type-ii or type-2 or type-iii or type-3))).mp.) or (((c3 or c-3 or serum-c3 or globulin-beta1-c or (globulin adj2 beta adj2 ("1c" or "1 c")) or ((complement? or component? or precursor?) adj1 "3")) adj4 glomerulo$) or ((C3GN or C3G or MPGN$3 or DDDMPGN$3 or 80295-41-6) and glomerulo$) or (dense adj3 deposit adj3 disease?) or ((membranoproliferat$ or membrano-proliferat$ or mesangiocapillar$ or mesangio-capillar$) adj4 (glomerulonephritis$ or glomerulo-nephriti$) adj4 (type-ii or type-2 or subendothelial$ or sub-endothelial$)) or (MPGN adj4 (type-ii or type-2 or type-iii or type-3))).ti,ab,kw,kf. [C3G TERMS] | 6663 |
| 2 | Network Meta-Analysis/ or Network Meta-Analysis as Topic/ or ((Meta-Analysis/ or Meta-Analysis as Topic/) and network.ti,ab,kf.) or network meta analysis.pt. [MA/NMA TERMS] | 40096 |
| 3 | meta-analysis.pt. or evidence report.jw. or ((meta-analy$ or metaanaly$) and network).mp,hw. or (met-analy$ or metanaly$ or meta regression$ or metaregression$ or ((indirect or indirect treatment? or mixed-treatment? or Bayesian or simulated treatment?) adj3 comparison$) or (multi$ adj3 treatment? adj3 comparison$) or (mixed adj3 treatment? adj3 (meta-analy$ or metaanaly$)) or (multi$ adj2 paramet$ adj2 evidence adj2 synthesis) or (multiparamet$ adj2 evidence adj2 synthesis) or (multi-paramet$ adj2 evidence adj2 synthesis)).ti,ab,kf. [SR / MA / ITC - CDA-AMC pragmatic filter, 2021 – MEDLINE – MODIFIED to focus on NMAs & ITCs] | 327349 |
| 4 | 2 or 3 [ALL MA/NMA/ITC TERMS] | 327349 |
| 5 | 1 and 4 | 5 |
| 6 | exp Animals/ not Humans/ [ANIMAL STUDIES ONLY - REMOVE - MEDLINE] | 11514538 |
| 7 | (address or biography or comment or editorial or "expression of concern" or festschrift or historical article or lecture or news or patient education handout or personal narrative or video-audio media or webcast or (letter not (letter and randomized controlled trial))).pt. [Opinion publications - Remove -MEDLINE] | 5313295 |
| 8 | 5 not (6 or 7) [ANIMAL STUDIES and OPINION PUBLICATIONS - REMOVED - MEDLINE] | 5 |
| **9** | **8 use ppezv [MEDLINE results]** | **4** |
| 10 | (membranoproliferative glomerulonephritis/ and (((c3 or c-3 or serum-c3 or globulin-beta1-c or (globulin adj2 beta adj2 ("1c" or "1 c")) or ((complement? or component? or precursor?) adj1 "3")) adj4 glomerulo$) or ((C3GN or C3G or MPGN$3 or DDDMPGN$3 or 80295-41-6) and glomerulo$)).mp.) or (exp complement component C3/ and (((C3GN or C3G or MPGN$3 or DDDMPGN$3 or 80295-41-6) and glomerulo$) or (dense adj3 deposit adj3 disease?) or ((membranoproliferat$ or membrano-proliferat$ or mesangiocapillar$ or mesangio-capillar$) adj4 (glomerulonephritis$ or glomerulo-nephriti$) adj4 (type-ii or type-2 or subendothelial$ or sub-endothelial$)) or (MPGN adj4 (type-ii or type-2 or type-iii or type-3))).mp.) or (((c3 or c-3 or serum-c3 or globulin-beta1-c or (globulin adj2 beta adj2 ("1c" or "1 c")) or ((complement? or component? or precursor?) adj1 "3")) adj4 glomerulo$) or ((C3GN or C3G or MPGN$3 or DDDMPGN$3 or 80295-41-6) and glomerulo$) or (dense adj3 deposit adj3 disease?) or ((membranoproliferat$ or membrano-proliferat$ or mesangiocapillar$ or mesangio-capillar$) adj4 (glomerulonephritis$ or glomerulo-nephriti$) adj4 (type-ii or type-2 or subendothelial$ or sub-endothelial$)) or (MPGN adj4 (type-ii or type-2 or type-iii or type-3))).ti,ab,kw,kf. [C3G TERMS] | 6663 |
| 11 | exp meta-analysis/ or exp "meta analysis (topic)"/ or (systematic review or meta-analysis).pt. or (met-analy$ or metanaly$ or meta regression$ or metaregression$ or ((indirect or indirect treatment? or mixed-treatment? or Bayesian or simulated treatment?) adj3 comparison$) or (multi$ adj3 treatment? adj3 comparison$) or (mixed adj3 treatment? adj3 (meta-analy$ or metaanaly$)) or (multi$ adj2 paramet$ adj2 evidence adj2 synthesis) or (multiparamet$ adj2 evidence adj2 synthesis) or (multi-paramet$ adj2 evidence adj2 synthesis)).ti,ab,kf. [SR / MA / ITC - CDA-AMC pragmatic filter, 2021 - Embase - MODIFIED to focus on NMAs & ITCs] | 897843 |
| 12 | 10 and 11 | 22 |
| 13 | (exp animal/ or exp animal experiment/ or exp animal model/ or nonhuman/ or exp vertebrate/) not (exp human/ or exp human experiment/) [ANIMAL STUDIES ONLY - REMOVE - EMBASE] | 13467685 |
| 14 | (editorial or note or short survey or tombstone).pt. or (letter.pt. not randomized controlled trial/) [OPINION PIECES REMOVE - Embase] | 5800174 |
| 15 | 12 not (13 or 14) [ANIMAL STUDIES and OPINION PUBLICATIONS - REMOVED - Embase] | 22 |
| 16 | conference abstract.pt. [CONFERENCE ABSTRACTS] | 5959752 |
| 17 | 15 and 16 [CONFERENCE ABSTRACTS ONLY] | 5 |
| 18 | limit 17 to yr="2023 -Current" | 0 |
| 19 | 15 not 16 [CONFERENCE ABSTRACTS REMOVED] | 17 |
| 20 | 18 or 19 [LAST 3 YRS OF ABSTRACTS RETAINED] | 17 |
| **21** | **20 use oemezd [Embase results]** | **10** |
| 22 | (((c3 or c-3 or serum-c3 or globulin-beta1-c or (globulin adj2 beta adj2 ("1c" or "1 c")) or ((complement? or component? or precursor?) adj1 "3")) adj4 glomerulo$) or ((C3GN or C3G or MPGN$3 or DDDMPGN$3 or 80295-41-6) and glomerulo$) or (dense adj3 deposit adj3 disease?) or ((membranoproliferat$ or membrano-proliferat$ or mesangiocapillar$ or mesangio-capillar$) adj4 (glomerulonephritis$ or glomerulo-nephriti$) adj4 (type-ii or type-2 or subendothelial$ or sub-endothelial$)) or (MPGN adj4 (type-ii or type-2 or type-iii or type-3))).ti,ab,kw,kf. [C3G TERMS] | 6532 |
| 23 | ((meta-analy$ or metaanaly$) and network).mp. or (met-analy$ or metanaly$ or meta regression$ or metaregression$ or ((indirect or indirect treatment? or mixed-treatment? or Bayesian or simulated treatment?) adj3 comparison$) or (multi$ adj3 treatment? adj3 comparison$) or (mixed adj3 treatment? adj3 (meta-analy$ or metaanaly$)) or (multi$ adj2 paramet$ adj2 evidence adj2 synthesis) or (multiparamet$ adj2 evidence adj2 synthesis) or (multi-paramet$ adj2 evidence adj2 synthesis)).ti,ab,kw. | 119116 |
| 24 | 22 and 23 | 1 |
| **25** | **24 use coch [CDSR results]** | **0** |
| 26 | 9 or 21 or 25 | 14 |
| **27** | **remove duplicates from 26** | **12** |

**Note**: The following databases were searched through the OvidSP platform on March 6, 2026: 1) MEDLINE(R) and Epub Ahead of Print; 2) In-Process, In-Data-Review & Other Non-Indexed Citations, Daily and Versions (1946 – March 5, 2026), and 3) Embase (1974 – March 4, 2026), and EBM Reviews Cochrane Database of Systematic Reviews (2005 – March 4, 2026).

**Appendix Table 3: List of excluded Studies**

| **#** | **Excluded Study** | **Exclusion Reason** |
| --- | --- | --- |
| 1 | Chauvet S, Berthaud R, Devriese M, Mignotet M, Martins PV, Robe-Rybkine T, Miteva MA, Gyulkhandanyan A, Ryckewaert A, Louillet F, Merieau E. Anti-factor B antibodies and acute postinfectious GN in children. Journal of the American Society of Nephrology. 2020 Apr 1;31(4):829-40. | Population (non-C3G population) |
| 2 | Ekrikpo UE, Obiagwu PN, Udo AI, Chukwuonye II, Noubiap JJ, Okpechi-Samuel US, et al. Prevalence and distribution of primary glomerular diseases in Africa: a systematic review and meta-analysis of observational studies. The Pan African medical journal. 2023;45:153. | Population (non-C3G population) |
| 3 | Fabrizi F, Lunghi G, Messa P, Martin P. Therapy of hepatitis C virus-associated glomerulonephritis: current approaches. Journal of nephrology. 2008;21(6):813-25. | Population (non-C3G population) |
| 4 | Gonzalez Suarez ML, Thongprayoon C, Hansrivijit P, Kovvuru K, Kanduri SR, Aeddula NR, et al. Treatment of C3 Glomerulopathy in Adult Kidney Transplant Recipients: A Systematic Review. Medical sciences (Basel, Switzerland). 2020;8(4). | Study Design (no ITC/NMA) |
| 5 | Kitchlu A, Jhaveri KD, Wadhwani S, Deshpande P, Harel Z, Kishibe T, et al. A Systematic Review of Immune Checkpoint Inhibitor-Associated Glomerular Disease. Kidney International Reports. 2021;6(1):66-77. | Population (non-C3G population) |
| 6 | Levin A. Management of membranoproliferative glomerulonephritis: Evidence-based recommendations. Kidney International, Supplement. 1999;55(70):S-46. | Study Design (no ITC/NMA) |
| 7 | Lin S, Zheng S, Dong W, Tian Y, Li H, Gao X, et al. Prevalence of histopathological subtypes associated with steroid-resistant nephrotic syndrome in children: a systematic review and meta-analysis. Frontiers in Immunology. 2025;16:1647608. | Population (non-C3G population) |
| 8 | Mallat SG, Itani HS, Abou-Mrad RM, Abou Arkoub R, Tanios BY. Rituximab use in adult primary glomerulopathy: Where is the evidence? Therapeutics and Clinical Risk Management. 2016;12:1317-27. | Study Design (no ITC/NMA) |
| 9 | Ozkok A, Yildiz A. Hepatitis C virus associated glomerulopathies. World Journal of Gastroenterology. 2014;20(24):7544-54. | Population (non-C3G population) |
| 10 | Sherman AC, Sherman KE. Extrahepatic Manifestations of Hepatitis C Infection: Navigating CHASM. Current HIV/AIDS Reports. 2015;12(3):353-61. | Population (non-C3G population) |
| 11 | Uffing A, Hullekes F, Riella LV, Hogan JJ. Recurrent glomerular disease after kidney transplantation diagnostic and management dilemmas. Clinical Journal of the American Society of Nephrology. 2021;16(11):1730-42. | Study Design (no ITC/NMA) |
| 12 | Wang TJ, Sheu WHH, Yang SS, Shu KH. Renal manifestations of hepatitis B virus infection. Journal of Internal Medicine of Taiwan. 2012;23(3):183-91. | Population (non-C3G population) |

**Appendix Table 4: Comparison of APPEAR‑C3G (iptacopan) and VALIANT (pegcetacoplan) inclusion and exclusion criteria**

| Characteristic | APPEAR-C3G (iptacopan)‑C3G (iptacopan) | VALIANT (pegcetacoplan) |
| --- | --- | --- |
| Inclusion criteria | - Male and female participants age ≥ 18 and ≤ 60 years at screening. - Diagnosis of C3G as confirmed by renal biopsy within 12 months prior to enrollment in adults and within 3 years in adolescents. - Prior to randomization, all participants must have been on a maximally recommended or tolerated dose of an angiotensin converting enzyme inhibitor (ACEI) or angiotensin receptor blocker (ARB) for at least 90 days. The doses of other antiproteinuric medications including mycophenolic acid, corticosteroids and mineralocorticoid receptor antagonists should be stable for at least 90 days prior to randomization. - Reduced serum C3 (defined as less than 0.85 x lower limit of the central laboratory normal range) at Screening. - UPCR ≥ 1.0 g/g sampled from the first morning void urine sample at Day -75 and Day -15. - Estimated GFR (using the CKD-EPI formula for ages ≥ 18 years and modified Schwartz formula for ages 12 to 17 years) or measured GFR ≥ 30 ml/min/1.73m2 at screening and Day -15. - Mandatory vaccination against Neisseria meningitidis and Streptococcus pneumoniae prior to the start of study treatment. - If not previously vaccinated or if a booster is required, vaccination against Haemophilus influenzae infections should be given, if available and according to local regulations, at least 2 weeks prior to the first study treatment administration. If study treatment has to start earlier than 2 weeks post vaccination, prophylactic antibiotic treatment should be initiated. | - Aged at least 18 years; where approved, adolescents (aged 12-17 years) weighing at least 30 kg may also be enrolled. - A diagnosis of primary C3G or IC-MPGN (with or without previous renal transplant). - Evidence of active renal disease, based on one or more of the following:   1. In adults or adolescents with a baseline renal biopsy (either one collected during screening or a historic biopsy collected within 28 weeks prior to randomization), at least 2+ C3c staining on the baseline renal biopsy.   2. In adolescents not providing a baseline renal biopsy, at least one of the following:   - Plasma sC5b-9 level above the upper limit of normal during screening   - Serum C3 below the LLN during screening   - Presence of an active urine sediment during screening, as evidenced by hematuria with at least 5 red blood cells (RBCs) per high-power field (HPF) and/or red blood cell casts on local or central microscopic analysis of urine.   - Presence of C3 nephritic factor within 6 months of screening, based on central laboratory results or medical history. - No more than 50% global glomerulosclerosis or interstitial fibrosis on the baseline biopsy for adult participants or adolescent participants providing a baseline biopsy. - At least 1 g/day of proteinuria on a screening 24-hour urine collection and a uPCR of at least 1000 mg/g in at least 2 first-morning spot urine samples collected during screening. - eGFR ≥30 mL/min/1.73 m2 calculated by the Chronic Kidney Disease-Epidemiology Collaboration creatinine equation for adults or the Bedside Schwartz equation for adolescents. - Stable regimen for C3G/IC-MPGN treatment, as described below:   - Angiotensin-converting enzyme inhibitor/, angiotensin receptor blocker, and/or sodium-glucose cotransporter-2 inhibitor therapy that is stable and optimized, in the opinion of the investigator, for at least 12 weeks prior to randomization   - Stable doses of other medications that can affect proteinuria (eg, steroids, mycophenolate mofetil, and/or other allowed immunosuppressants that the participant is receiving for treatment of C3G or IC-MPGN) for at least 812 weeks prior to the baseline renal biopsy and randomization.   - If a participant is on prednisone (or other systemic corticosteroid) for C3G or IC-MPGN treatment, the dosage is stable and no higher than 20 mg/day (or equivalent dosage of a corticosteroid other than prednisone) for at least 12 weeks prior to randomization. - Have received vaccinations against S pneumoniae, N meningitidis (types A, C, W, Y, and B), and H influenzae (type B) within 5 years prior to randomization or agree to receive vaccinations during screening. |
| Exclusion criteria | - Participants who have received any cell or organ transplantation, including a kidney transplantation. - Rapidly progressive crescentic glomerulonephritis defined as a 50% decline in the eGFR within 3 months with renal biopsy findings of glomerular crescent formation seen in at least 50% of glomeruli. - Renal biopsy showing interstitial fibrosis/tubular atrophy (IF/TA) of more than 50% - Monoclonal gammopathy of undetermined significance (MGUS) confirmed by the measurement of serum free light chains or other investigation as per local standard of care. - Participants with an active systemic bacterial, viral or fungal infection within 14 days prior to study treatment administration - The presence of fever ≥ 38°C (100.4°F) within 7 days prior to study treatment administration. - A history of recurrent invasive infections caused by encapsulated organisms, e.g., N. meningitidis and S. pneumoniae. - The use of inhibitors of complement factors (e.g., Factor B, Factor D, C3 inhibitors, anti C5 antibodies, C5a receptor antagonists) within 6 months prior to the Screening visit. - The use of immunosuppressants (except mycophenolic acids), cyclophosphamide or systemic corticosteroids at a dose >7.5 mg/day (or equivalent for a similar medication) within 90 days of study drug administration. - Acute post-infectious glomerulonephritis at screening based upon the opinion of the investigator. | - Previous exposure to pegcetacoplan. - C3G/IC-MPGN secondary to another condition (eg, infection, malignancy, monoclonal gammopathy, a systemic autoimmune disease such as systemic lupus erythematosus, chronic antibody-mediated rejection, or a medication), in the opinion of the investigator. - Current or prior diagnosis of human immunodeficiency virus (HIV), hepatitis B (HBV), or hepatitis C (HCV) infection or positive serology during screening that is indicative of infection with any of these viruses. - Body weight greater than 100 kg at screening. - Hypersensitivity to pegcetacoplan or to any of the excipients. - History of meningococcal disease. - Malignancy, except for the following: - Cured basal or squamous cell skin cancer - Curatively treated in situ disease - Malignancy-free and off treatment for ≥5 years - Severe infection (eg, requiring IV antibiotic therapy) within 14 days prior to the first dose of pegcetacoplan. - An absolute neutrophil count <1000 cells/mm3 at screening. - Use of rituximab, belimumab, or any approved or investigational anticomplement therapy other than pegcetacoplan within 5 half-lives of that product prior to the screening period. - Female participants who are pregnant or who are currently breastfeeding and are unwilling to discontinue for the duration of the study and for at least 90 days after the final dose of study drug. - Presence or suspicion of severe infection during the screening period (including but not limited to recurrent or chronic infections) that, in the opinion of the investigator, may place the participant at unacceptable risk by study participation. - Known or suspected hereditary fructose intolerance. |

**Appendix Figure 1: PRISMA flow diagram of the study selection process.** PRISMA flow diagram for the conducted SLR, including number of studies identified via searches of databases, registers and other sources
