# Supplementary material for: Methodological pitfalls in indirect treatment comparisons: insights from a recent systematic review and analysis for C3 glomerulopathy
Source: J Comp Eff Res. 2026 Aug 9;15(9):e260054. doi: 10.57264/cer-2026-0054 (PMC13556872; doi:10.57264/cer-2026-0054)
Supplement: Supplementary file 2 [file cer-15-260054-s2.docx]

# PRISMA 2020 Main Checklist

| **Topic** | **No.** | **Item** | **Location where item is reported** |
| --- | --- | --- | --- |
| **TITLE** |  |  |  |
| **Title** | 1 | Identify the report as a systematic review. | Title page |
| **ABSTRACT** |  |  |  |
| **Abstract** | 2 | See the PRISMA 2020 for Abstracts checklist |  |
| **INTRODUCTION** |  |  |  |
| **Rationale** | 3 | Describe the rationale for the review in the context of existing knowledge. | Methods, p. 5 (Systematic Literature Review to identify published ITCs in C3G) |
| **Objectives** | 4 | Provide an explicit statement of the objective(s) or question(s) the review addresses. | Methods, p.5 (Critical Appraisal of Comparative ITCs identified in SLR) |
| **METHODS** |  |  |  |
| **Eligibility criteria** | 5 | Specify the inclusion and exclusion criteria for the review and how studies were grouped for the syntheses. | Methods, p. 5 (Systematic Literature Review to identify published ITCs in C3G); Appendix Table 1 |
| **Information sources** | 6 | Specify all databases, registers, websites, organisations, reference lists and other sources searched or consulted to identify studies. Specify the date when each source was last searched or consulted. | Methods, p. 5 (Systematic Literature Review to identify published ITCs in C3G) |
| **Search strategy** | 7 | Present the full search strategies for all databases, registers and websites, including any filters and limits used. | Methods, p. 5 (Systematic Literature Review to identify published ITCs in C3G); Appendix Table 2 |
| **Selection process** | 8 | Specify the methods used to decide whether a study met the inclusion criteria of the review, including how many reviewers screened each record and each report retrieved, whether they worked independently, and if applicable, details of automation tools used in the process. | Methods, p. 6 (Systematic Literature Review to identify published ITCs in C3G) |
| **Data collection process** | 9 | Specify the methods used to collect data from reports, including how many reviewers collected data from each report, whether they worked independently, any processes for obtaining or confirming data from study investigators, and if applicable, details of automation tools used in the process. | Methods, p. 6 (Systematic Literature Review to identify published ITCs in C3G) |
| **Data items** | 10a | List and define all outcomes for which data were sought. Specify whether all results that were compatible with each outcome domain in each study were sought (e.g. for all measures, time points, analyses), and if not, the methods used to decide which results to collect. | Appendix Table 1 |
|  | 10b | List and define all other variables for which data were sought (e.g. participant and intervention characteristics, funding sources). Describe any assumptions made about any missing or unclear information. | Appendix Table 1 |
| **Study risk of bias assessment** | 11 | Specify the methods used to assess risk of bias in the included studies, including details of the tool(s) used, how many reviewers assessed each study and whether they worked independently, and if applicable, details of automation tools used in the process. | Not applicable. The SLR only identified a poster/abstract, thus a risk of bias assessment was not required and details on methods were there excluded. |
| **Effect measures** | 12 | Specify for each outcome the effect measure(s) (e.g. risk ratio, mean difference) used in the synthesis or presentation of results. | The remaining sections (12-15) are not applicable in this manuscript as only one poster/abstract was identified in the SLR. The manuscript instead focuses on a critical appraisal of the individual methods used in the reported ITC and does not include a synthesis of any data and does not report any outcome effect data. The manuscript also comments on the certainty of the reported ITC evidence in the discussion section of the manuscript, though this serves the objectives of our research questions rather than addressing the certainty of evidence related to a summary of outcome data obtained from identified studies. |
| **Synthesis methods** | 13a | Describe the processes used to decide which studies were eligible for each synthesis (e.g. tabulating the study intervention characteristics and comparing against the planned groups for each synthesis (item 5)). |  |
|  | 13b | Describe any methods required to prepare the data for presentation or synthesis, such as handling of missing summary statistics, or data conversions. |  |
|  | 13c | Describe any methods used to tabulate or visually display results of individual studies and syntheses. |  |
|  | 13d | Describe any methods used to synthesize results and provide a rationale for the choice(s). If meta-analysis was performed, describe the model(s), method(s) to identify the presence and extent of statistical heterogeneity, and software package(s) used. |  |
|  | 13e | Describe any methods used to explore possible causes of heterogeneity among study results (e.g. subgroup analysis, meta-regression). |  |
|  | 13f | Describe any sensitivity analyses conducted to assess robustness of the synthesized results. |  |
| **Reporting bias assessment** | 14 | Describe any methods used to assess risk of bias due to missing results in a synthesis (arising from reporting biases). |  |
| **Certainty assessment** | 15 | Describe any methods used to assess certainty (or confidence) in the body of evidence for an outcome. |  |
| **RESULTS** |  |  |  |
| **Study selection** | 16a | Describe the results of the search and selection process, from the number of records identified in the search to the number of studies included in the review, ideally using a flow diagram. | Results, p.8 (Systematic Literature Review of Published ITCs in C3G); Appendix Figure 1 |
|  | 16b | Cite studies that might appear to meet the inclusion criteria, but which were excluded, and explain why they were excluded. | Appendix Table 3 |
| **Study characteristics** | 17 | Cite each included study and present its characteristics. | Results, p.8 (Systematic Literature Review of Published ITCs in C3G) |
| **Risk of bias in studies** | 18 | Present assessments of risk of bias for each included study. | Not applicable. The SLR only identified a poster/abstract, thus a risk of bias assessment was not required and details on methods were there excluded. |
| **Results of individual studies** | 19 | For all outcomes, present, for each study: (a) summary statistics for each group (where appropriate) and (b) an effect estimate and its precision (e.g. confidence/credible interval), ideally using structured tables or plots. | The remaining sections (19-22) are not applicable in this manuscript as only one poster/abstract was identified in the SLR. The manuscript instead focuses on a critical appraisal of the individual methods used in the reported ITC and does not include a synthesis of any data and does not report any outcome effect data. The manuscript also comments on the certainty of the reported ITC evidence in the discussion section of the manuscript, though this serves the objectives of our research questions rather than addressing the certainty of evidence related to a summary of outcome data obtained from identified studies. |
| **Results of syntheses** | 20a | For each synthesis, briefly summarise the characteristics and risk of bias among contributing studies. |  |
|  | 20b | Present results of all statistical syntheses conducted. If meta-analysis was done, present for each the summary estimate and its precision (e.g. confidence/credible interval) and measures of statistical heterogeneity. If comparing groups, describe the direction of the effect. |  |
|  | 20c | Present results of all investigations of possible causes of heterogeneity among study results. |  |
|  | 20d | Present results of all sensitivity analyses conducted to assess the robustness of the synthesized results. |  |
| **Reporting biases** | 21 | Present assessments of risk of bias due to missing results (arising from reporting biases) for each synthesis assessed. |  |
| **Certainty of evidence** | 22 | Present assessments of certainty (or confidence) in the body of evidence for each outcome assessed. |  |
| **DISCUSSION** |  |  |  |
| **Discussion** | 23a | Provide a general interpretation of the results in the context of other evidence. | Discussion, p.11 (Summary of main findings) |
|  | 23b | Discuss any limitations of the evidence included in the review. | Discussion, p.11 (Summary of main findings) |
|  | 23c | Discuss any limitations of the review processes used. | Discussion, p.11 (Summary of main findings) |
|  | 23d | Discuss implications of the results for practice, policy, and future research. | Discussion, p11 (Implications for clinical, market access, and HTA decision‑making) |
| **OTHER INFORMATION** |  |  |  |
| **Registration and protocol** | 24a | Provide registration information for the review, including register name and registration number, or state that the review was not registered. | Methods, p.5 (Systematic Literature Review to identify published ITCs in C3G) |
|  | 24b | Indicate where the review protocol can be accessed, or state that a protocol was not prepared. | Methods, p.5 (Systematic Literature Review to identify published ITCs in C3G) |
|  | 24c | Describe and explain any amendments to information provided at registration or in the protocol. | Not applicable given that the protocol was not produced or registered |
| **Support** | 25 | Describe sources of financial or non-financial support for the review, and the role of the funders or sponsors in the review. | Title Page |
| **Competing interests** | 26 | Declare any competing interests of review authors. | Title Page |
| **Availability of data, code and other materials** | 27 | Report which of the following are publicly available and where they can be found: template data collection forms; data extracted from included studies; data used for all analyses; analytic code; any other materials used in the review. | Methods, p.5 (Systematic Literature Review to identify published ITCs in C3G) |

# PRIMSA Abstract Checklist

| **Topic** | **No.** | **Item** | **Reported?** |
| --- | --- | --- | --- |
| **TITLE** |  |  |  |
| **Title** | 1 | Identify the report as a systematic review. | Yes |
| **BACKGROUND** |  |  |  |
| **Objectives** | 2 | Provide an explicit statement of the main objective(s) or question(s) the review addresses. | Yes |
| **METHODS** |  |  |  |
| **Eligibility criteria** | 3 | Specify the inclusion and exclusion criteria for the review. | Yes |
| **Information sources** | 4 | Specify the information sources (e.g. databases, registers) used to identify studies and the date when each was last searched. | No; The main focus of the manuscript is the critical appraisal, and given restrictions on abstract length, this was omitted for brevity but is included in the methods section. |
| **Risk of bias** | 5 | Specify the methods used to assess risk of bias in the included studies. | Not applicable. The SLR only identified a poster/abstract, thus a risk of bias assessment was not required and details on methods were there excluded. |
| **Synthesis of results** | 6 | Specify the methods used to present and synthesize results. | Yes |
| **RESULTS** |  |  |  |
| **Included studies** | 7 | Give the total number of included studies and participants and summarise relevant characteristics of studies. | Yes |
| **Synthesis of results** | 8 | Present results for main outcomes, preferably indicating the number of included studies and participants for each. If meta-analysis was done, report the summary estimate and confidence/credible interval. If comparing groups, indicate the direction of the effect (i.e. which group is favoured). | Yes |
| **DISCUSSION** |  |  |  |
| **Limitations of evidence** | 9 | Provide a brief summary of the limitations of the evidence included in the review (e.g. study risk of bias, inconsistency and imprecision). | No; The main focus of the manuscript is the critical appraisal, and given restrictions on abstract length, this was omitted for brevity. However, the abstract does highlight limitations with the identified ITC poster |
| **Interpretation** | 10 | Provide a general interpretation of the results and important implications. | Yes |
| **OTHER** |  |  |  |
| **Funding** | 11 | Specify the primary source of funding for the review. | No; Included in the title page |
| **Registration** | 12 | Provide the register name and registration number. | No; protocol was not registered |

*From:* Page MJ, McKenzie JE, Bossuyt PM, Boutron I, Hoffmann TC, Mulrow CD, et al. The PRISMA 2020 statement: an updated guideline for reporting systematic reviews. MetaArXiv. 2020, September 14. DOI: 10.31222/osf.io/v7gm2. For more information, visit: <www.prisma-statement.org>
